# Immune Cell Infiltrate in Chronic-Active Antibody-Mediated Rejection

**DOI:** 10.3389/fimmu.2019.03106

**Published:** 2020-02-04

**Authors:** Kasia A. Sablik, Ekaterina S. Jordanova, Noelle Pocorni, Marian C. Clahsen-van Groningen, Michiel G. H. Betjes

**Affiliations:** ^1^Department of Nephrology and Transplantation, Erasmus Medical Center, Rotterdam, Netherlands; ^2^Department of Gynecology, Center for Gynecologic Oncology, Amsterdam UMC, Amsterdam, Netherlands; ^3^Department of Pathology, Erasmus Medical Center, Rotterdam, Netherlands

**Keywords:** kidney transplantation, pathology, chronic rejection in renal transplant, antibody-mediated allograft rejection, immune cell

## Abstract

**Background:** Little is known about immune cell infiltrate type in the kidney allograft of patients with chronic-active antibody-mediated rejection (c-aABMR).

**Methods:** In this study, multiplex immunofluorescent staining was performed on 20 cases of biopsy-proven c-aABMR. T-cell subsets (CD3, CD8, Foxp3, and granzyme B), macrophages (CD68 and CD163), B cells (CD20), and natural killer cells (CD57) were identified and counted in the glomeruli (cells/glomerulus) and the tubulointerstitial (TI) compartment [cells/high-power field (HPF)].

**Results:** In the glomerulus, T cells and macrophages were the dominant cell types with a mean of 5.5 CD3^+^ cells/glomerulus and 4 CD68^+^ cells/glomerulus. The majority of T cells was CD8^+^ (62%), and most macrophages were CD68^+^CD163^+^ (68%). The TI compartment showed a mean of 116 CD3^+^ cells/HPF, of which 54% were CD8^+^. Macrophage count was 21.5 cells/HPF with 39% CD68^+^CD163^+^. CD20^+^ cells were sporadically present in glomeruli, whereas B-cell aggregates in the TI compartment were frequently observed. Natural killer cells were rarely identified. Remarkably, increased numbers of CD3^+^FoxP3^+^ cells in the TI compartment were associated with decreased graft survival (*p* = 0.004).

**Conclusions:** Renal allograft biopsies showing c-aABMR show a predominance of infiltrating CD8^+^ T cells, and increased numbers of interstitial FoxP3^+^ T cells are associated with inferior allograft survival.

## Introduction

The renal biopsies of patients with chronic-active antibody-mediated rejection (c-aABMR) have defining histomorphological lesions (1). These characteristic lesions include double contours of the glomerular basement membrane (transplant glomeropathy) and/or severe peritubular capillary basement membrane multilayering. In addition, evidence of current or recent antibody interaction with the vascular endothelium is provided by either linear C4d staining in peritubular capillaries or moderate microvascular inflammation [(g + ptc) ≥ 2] (2). The presence of donor-specific antibodies underlies c-aABMR, but the influx of inflammatory cells and rate of loss of renal function may vary substantially.

Previously, different studies have reported on the inflammatory cell types involved in renal allograft rejection. Hidalgo et al. provided evidence for a possible effector role for natural killer (NK) cells in endothelial injury during ABMR (3). In addition, ABMR is associated with both glomerular and interstitial monocyte infiltration (4–7). Mengel et al. showed the importance of inflammatory infiltrates in protocol biopsies, as they were independently associated with allograft survival, regardless of their location (8). Furthermore, activated cytotoxic T cells are known to play an important role in the inflammatory activity and destruction of allograft tissue (9). The increased presence of cytotoxic granules such as granzyme B have been associated with acute rejection (10–12). Similarly, FoxP3^+^ T cells [T regulatory cells (Tregs)] have been identified as potential crucial players in inflammatory disease and allograft rejection (13–15).

However, the majority of studies has focused on acute rejection (both TCMR and ABMR), and there is currently a lack of data on the presence and clinical relevance of inflammatory cells in renal allografts of patients with c-aABMR.

In the current study, we used multiplex immunofluorescent (IF) staining to assess the inflammatory cell composition in the renal allograft biopsy of patients with c-aABMR. We report on the presence of various T-cell subtypes, B cells, NK cells, and myeloid cells in both glomeruli and the tubulointerstitial (TI) compartment. In addition, the association between location and quantity of the different inflammatory cells with allograft survival was analyzed.

## Materials and Methods

### Study Population and Material

Twenty patients with biopsy-proven c-aABMR between 2010 and 2014 were included in this retrospective study. Sufficient formalin-fixed paraffin-embedded (FFPE) material after diagnostic process was needed to be included for further analysis. Renal transplant biopsies were all for cause and deemed necessary by the treating nephrologist due to a unexplained decline in renal allograft function with or without new onset proteinuria. All stained slides were re-evaluated and scored by two blinded pathologists according to the Banff 15 criteria (16). Donor-specific antibodies (DSA) were retrospectively assessed for all patients. The study was approved by the Medical Ethical Committee of the Erasmus MC (MEC-2019-0308).

### Staining

For routine diagnostic analysis, 3 μm sections were cut from the FFPE tissue and stained by H&E, periodic acid–Schiff–diastase, and Jones according to standardized diagnostic protocol at the Department of Pathology at the Erasmus Medical Center. Immunofluorescence staining was performed on snap-frozen tissue sections [immunoglobulin G (IgG), IgA, IgM, C3, C1q, kappa, and lambda] to exclude immune-complex-mediated disease.

**Figure 1:**
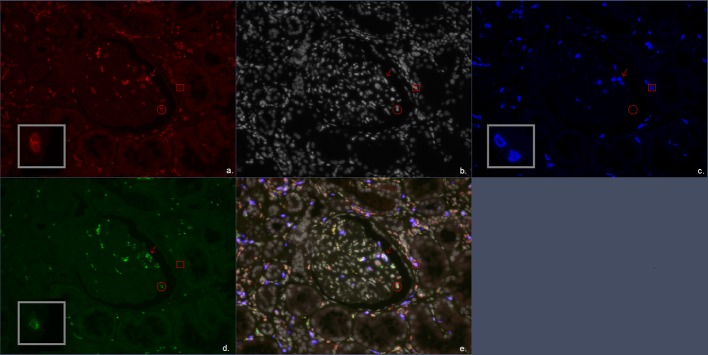
Multiplex immunofluorescent (IF) staining image of the antibody combination CD3, CD8, and granzyme B. **(a)** Red, CD3; **(b)** white, DAPI; **(c)** blue, CD8; **(d)** green, granzyme B; **(e)** combination of CD3, CD8, granzyme B, and DAPI. The arrow marks a CD3^+^CD8^+^granzyme B^+^ cell representing a CD8^+^ cytotoxic T cell. The circle encloses a CD3^+^granzyme B^+^ cell representing a CD4^+^ cytotoxic T cell. The square encloses a CD3^+^CD8^+^ cell representing a CD8^+^ T cell.

**Figure 2:**
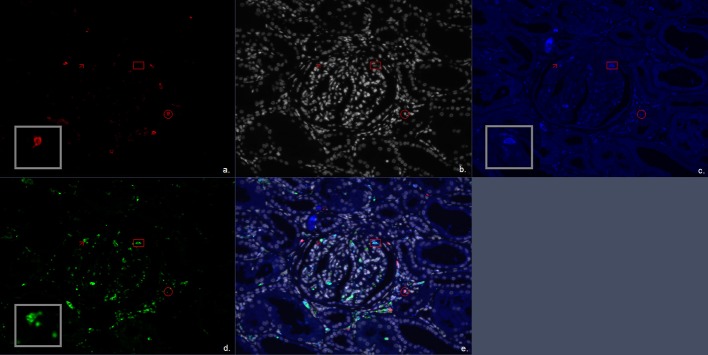
Multiplex immunofluorescent (IF) staining image of the antibody combination CD20, CD68, and CD163. **(a)** Red, CD20; **(b)** white, DAPI; **(c)** blue, CD163; **(d)** green, CD68; **(e)** combination of CD20, CD68, CD163, and 4′,6-diamidino-2-phenylindole (DAPI). The arrow marks a CD68^+^ cell representing a single positive CD68 macrophage. The circle encloses a CD20^+^ cell representing a B cell. The square encloses a CD68^+^CD163^+^ cell representing a double positive M2 macrophage subtype.

**Figure 3:**
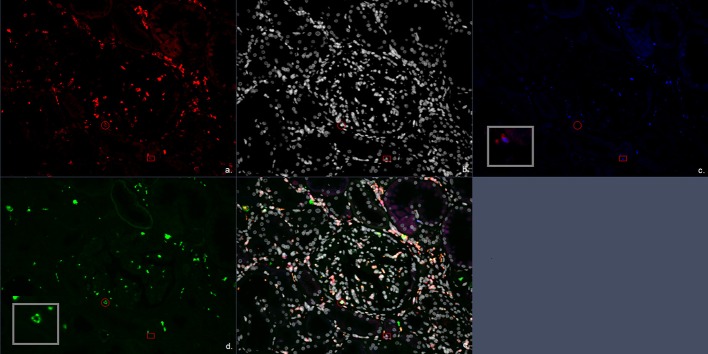
Multiplex immunofluorescent (IF) staining image of the antibody combination CD3, CD57, and FoxP3. **(a)** Red, CD3; **(b)** white, DAPI; **(c)** blue, FoxP3; **(d)** green, CD57; **(e)** combination of CD3, CD57, FoxP3, and 4′,6-diamidino-2-phenylindole (DAPI). The circle encloses a CD3^+^CD57^+^ cell representing a CD3^+^CD57^+^ cytotoxic T cells. The square encloses a CD3^+^FoxP3^+^ cell representing a FoxP3^+^ T regulatory cell.

Additional quadruple multiplex IF staining was performed on 4 μm FFPE sections. Multiplex IF staining was performed using the antibody combination of CD3, CD8, and granzyme B for CD4^+^ T cells, CD8^+^ cytotoxic T cells both with or without granzyme B presence (Figure 1). Second, CD20 was used as a B-cell marker, and for the identification of macrophages, the pan macrophage marker CD68 was used in combination with CD163 to distinguish the M2 macrophage subtype (profibrotic) (Figure 2). Lastly, a combination of CD3, FoxP3, and CD57 was used to identify CD3^+^FoxP3^+^ regulatory T cells and NK cells (Figure 3). 4′,6-Diamidino-2-phenylindole (DAPI) was used in all stainings to visualize the nuclei. Multiplex IF was performed as previously described by Punt et al. (17). In brief, after deparaffinization, ethylenediaminetetraacetic acid (pH 9) antigen retrieval was performed in a microwave. Next, three different combinations of primary antibodies were applied to the slides. The first mixture consisted of the antibodies anti-CD3 (rabbit polyclonal, ab828; Abcam), anti-CD8 (mouse monoclonal IgG2b, 4B11; Novocastra), and anti-Granzyme B (mouse monoclonal IgG2a, GRB-7, DAKO). The second mixture consisted of the antibodies anti-CD3 (rabbit polyclonal, ab828; Abcam), anti-FoxP3 (mouse monoclonal IgG1, 236A/E7, Abcam), and anti-CD57 (mouse monoclonal IgM, HNK-1; developed in Leiden University Medical Center). The third mixture comprised the antibodies anti-CD20 (mouse-IgG2a, L2G, Ventana), anti-CD163 (rabbit, ab100909, Abcam), and anti-CD68 (mouse-IgG1, 123C3, Ventana). The first combination was visualized using a combination of fluorescent antibody conjugates (Molecular Probes, ThermoFisher): goat antirabbit IgG–Alexa Fluor 546, goat antimouse IgG2b–Alexa Fluor 647, and goat antimouse IgG2a–Alexa Fluor 488. The second combination was visualized using goat antirabbit IgG–Alexa Fluor 546, goat antimouse IgG1–Alexa Fluor 647, and goat antimouse IgM–Alexa Fluor 488. The third combination was visualized using goat antimouse IgG2a–Alexa Fluor 546, antirabbit IgG–Alexa Fluor 647, and goat antimouse IgG1–Alexa Fluor 488. The panels were optimized using normal human tonsil and normal renal cortical tissue.

### Detection and Analysis

Images of the slides were taken for further analysis on a confocal laser scanning microscope in multitrack setting (Zeiss LSM700, Zeiss, Jena, Germany). An LCI Plan-Neofluar 25×/0.8 Imm Korr DIC objective (Zeiss) was used.

If possible, tissue sections were scanned in their entirety. For the glomerular cell counts, all available glomeruli per silver-stained tissue were included. The glomerular compartment cell counts were represented as the average number of whole cells per glomeruli. Tubulo-interstitial compartment cell counts were represented as the average number of whole cells per high-power field (HPF, 40×). A blinded and random selection of 3 HPF was analyzed for the TI compartment cell counts. All cells were counted using the Zen 2.3 SP1 software.

### Statistical Analysis

Normally distributed data are expressed as mean ± SD, non-normally distributed data as median (interquartile range). All statistical analysis were performed using Graphpad Prism 6 and SPSS software version 24. Statistical significance was calculated using unpaired *t*-test for continuous variables, Mann–Whitney *U*-test for ordinal variables, and chi-squared or Fisher exact test for categorical variables. A *P* < 0.05 was considered statistically significant.

Graft survival curves, starting at time of c-aABMR diagnosis, were censored for death with functioning graft and analyzed by Kaplan–Meier with log-rank test. For the analysis of association of inflammatory cells with allograft survival, both the glomerular and TI compartment cell count were divided dichotomously based on the mean cell count.

## Results

### Baseline Characteristics

**Figure 4:**
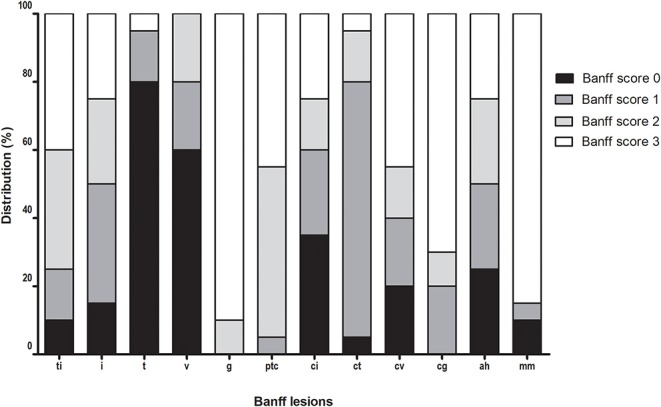
Distribution of Banff lesions at time of chronic-active antibody-mediated rejection (c-aABMR) diagnosis.

Clinical and histological characteristics of the included patients are shown in Table 1 and Figure 4. The mean age of the patients was 54 years at the time of transplant biopsy. Mean time point of biopsy post-transplantation was 3.6 years. Patients were predominantly treated with an immunosuppressive regimen using a combination of calcineurin inhibitors (mainly tacrolimus, 80%) and mycophenolate mofetil (90%). Mean follow-up was 3.4 years (range, 0.7–8.3 years) or until graft failure (either retransplantation or return to dialysis). Two patients died with a functioning graft during follow-up.

**Table 1:** Main clinical features at time of chronic-active antibody-mediated rejection (c-aABMR) diagnosis.

		***N* = 20**
Recipients age, years (IQR)		54 (44–63)
Male, *n* (%)		14 (70)
Donor age, years (IQR)		52 (40–59)
Prior transplantation, *n* (%)		7 (35)
Living donation, *n* (%)		13 (65)
HLA mismatch, median (IQR)		3 (2–4)
Time post-transplantation, years (IQR)		3.6 (1.8–7.5)
eGFR, ml/min/1.73 m^2^ (IQR)		29 (24–38)
Proteinuria, g/L (IQR)		0.75
DSA positive, *n* (%)		9 (45)^*^
	HLA class I	2
	HLA class II	8
C4d positive, *n* (%)		10 (50)
Renal disease, *n* (%)	Diabetic nephropathy	5 (25)
	Hypertensive nephropathy	2 (10)
	Reflux nephropathy	2 (10)
	Chronic pyelonephritis	2 (10)
	Cystic kidney disease	2 (10)
	Other	7 (35)
Immunosuppressive therapy, *n* (%)	Tacrolimus	16 (80)
	MMF	18 (90)
	Corticosteroids	9 (45)
	Other	1 (5)

* *One patient had both HLA class I and HLA class II DSA present*.

Forty-five percent of patients were DSA positive at the time of biopsy, 50% showed linear positive C4d staining of the peritubular capillaries, and all biopsies showed microvascular inflammation. Furthermore, all patients had severe double contours of the glomerular basement membrane (cg) present in their biopsy with moderate to severe chronic damage [interstitial fibrosis and tubular atrophy (IFTA)].

### CD3^+^, CD8^+^, Granzyme B^+^ T Cells

**Figure 5:**
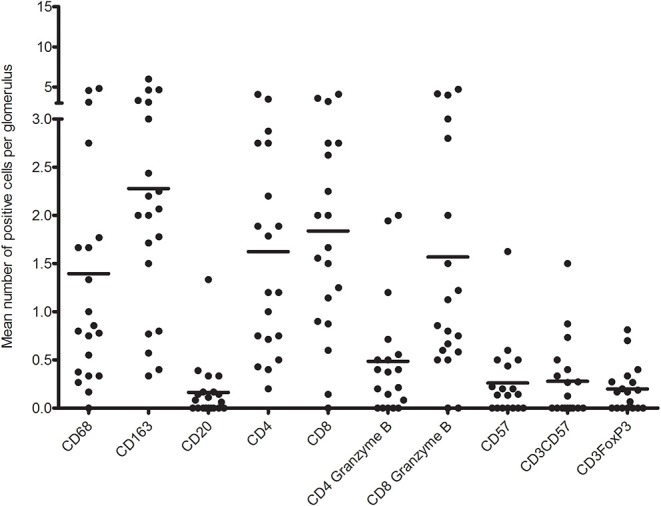
Distribution of mean number of inflammatory immune cells per glomerulus per biopsy.

**Figure 6:**
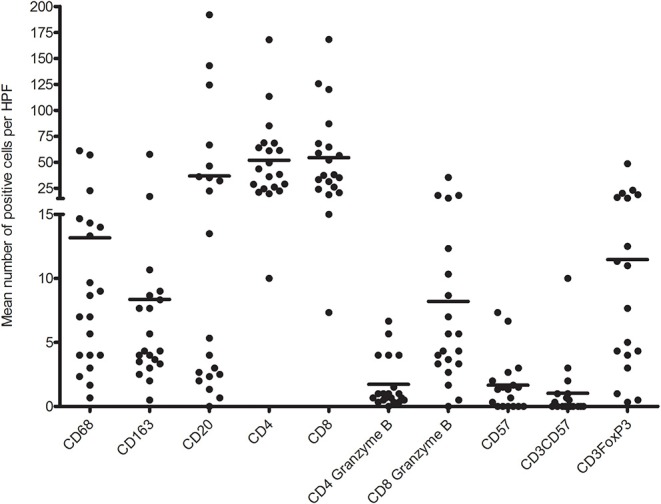
Distribution of mean number of inflammatory immune cells per high-power field (HPF) per biopsy.

First, we analyzed the number of glomerular T cell (CD3^+^ T cells), CD8^+^ T cells (CD3^+^CD8^+^ T cells), CD4^+^ T cells (CD3^+^CD8^−^ T cells), CD8^+^granzyme B^+^ T cells, and CD4^+^granzyme B^+^ T cells by quadruple immunostaining. Figure 5 shows the distribution as well as the mean number of inflammatory cells per glomerulus per biopsy. A mean total of 5.5 CD3^+^ cells were present per glomerulus with a range of 0.8–9.6 CD3^+^ cells. CD8^+^ T cells were predominant, making up 61.7% of CD3^+^ T cells with a mean of 3.4 cells per glomerulus. A minority of CD8^+^ T cells (46%) and CD4^+^ T cell (23%) showed cytotoxic potential as measured by coexpression of granzyme B. Inflammatory cell counts were then performed for the TI compartment (Figure 6). Although substantial variations were observed in the number of infiltrating cells among the biopsies, large areas of CD3^+^ T cells were abundantly present within the TI compartment. The TI showed a mean CD3^+^ cell count of 116.2 per HPF. The majority of CD3^+^ T cells present in the TI represented CD8^+^ T cells (53.9%) with an average cell count of 62.6 per HPF followed by CD4^+^ T cells (46.2%) with 53.6 positive cells per HPF. In contrast to the glomeruli, there were hardly any CD8^+^ granzyme B^+^ T cells (7% of total CD3^+^ T cells, 8.2 cells per HPF) and CD4^+^ granzyme B^+^ T cells (1.5% of total CD3^+^ T cells, 1.7 cells per HPF) present.

No significant association was found for the presence of CD3^+^ T cells and the T-cell subsets in the glomeruli and TI compartment with regard to allograft survival or DSA presence.

### CD3^+^, FoxP3^+^ T Cells, and CD57^+^ NK Cells

The second quadruple multiplex IF staining identified CD57^+^ NK cells (CD3^−^), CD3^+^FoxP3^+^ regulatory T cells, and CD3^+^CD57^+^ T cells.

The glomeruli showed low counts of CD57^+^ and CD3^+^CD57^+^ cells in this multiplex IF staining (Figure 5). Almost 40% of biopsies showed no presence of CD57^+^ or CD3^+^FoxP3^+^ cells in the glomeruli. The remaining biopsies showed a mean of 0.67 CD57^+^, 0.56 CD3^+^CD57^+^, and 0.32 FoxP3^+^ cells per glomerulus. CD3^+^CD57^+^ cytotoxic T cells accounted for 6.7% of CD3^+^ cells in the glomerulus.

**Figure 7:**
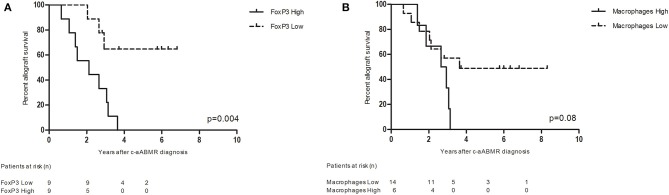
**(A)** Renal allograft survival after chronic-active antibody-mediated rejection (c-aABMR) diagnosis in relation to CD3^+^ FoxP3^+^ cell presence in the tubulointerstitial compartment; **(B)** renal allograft survival after c-aABMR diagnosis in relation to overall macrophage (CD68^+^ and CD163^+^) presence in the tubulointerstitial compartment.

The TI compartment, however, showed a much higher presence of CD3^+^FoxP3^+^ T cells with an average of 11.5 cells per HPF. These cells accounted for 8.5% of the CD3^+^ cells. A clear distinction into two groups was noticeable within the biopsies for CD3^+^FoxP3^+^ T cells (high vs. low mean cell count). Fifty percent of biopsies had a high mean cell count of 19.6 positive cells per HPF vs. a low mean cell count of 3.4 positive cells per HPF. The increased presence of CD3^+^FoxP3^+^ T cells was significantly associated with a decreased allograft survival (Figure 7A). Patients with high CD3^+^FoxP3^+^ T cell rates had a graft survival of 2.1 vs. 5.3 years in the patients with low FoxP3 presence (*p* = 0.004).

Similar to what was observed for the glomeruli, the CD57^+^ cell count in the TI was low with a mean of 1.7 cells per HPF and CD3^+^CD57^+^ T cells accounted for only 0.8% of CD3^+^ cells in this compartment.

### CD68^+^, CD163^+^ Macrophages, and CD20^+^ B Cells

The third multiplex IF staining panel included markers for macrophages (CD68^+^), M2 macrophages (CD68^+^CD163^+^), and B cells (CD20^+^).

CD20^+^ cells were sporadically present in glomeruli with a mean number of 0.16 positive cells per glomerulus. Interestingly, 45% of biopsies hardly contained any B cells in the glomeruli. The macrophages (CD68^+^ cells) represented mean number of almost four cells per glomerulus. The majority (68%) was CD68^+^CD163^+^ with a mean positive cell count of 2.3 per glomerulus. A scattered distribution of macrophages was visible with ranges of 0–6 positive cells per glomerulus. No significant association with graft function or DSA presence was found for macrophage or B cell presence in the glomeruli (data not shown).

In contrast to the glomeruli, the TI compartment showed a higher percentage of CD68^+^ cells (61%) with a mean positive cell count of 13.2 per HPF. CD68^+^CD163^+^ macrophages accounted for 39% of macrophages with a mean of 8.4 positive cells per HPF. The presence of total CD68^+^ and CD68^+^CD163^+^ macrophages in the TI compartment showed a near significant inverse association with graft survival (*p* = 0.08) (Figure 7B).

Furthermore, a mean number of 36.8 positive CD20 cells was counted in the tubulointerstitium. However, as with the CD3^+^FoxP3^+^ T cells, a clear distribution into two groups was visible. Forty-five percent of the biopsies were found to present CD20^+^ cells in nodular formation with a mean of 74.5 CD20-positive cells per HPF. The remaining biopsies reached a mean of 3.4 CD20^+^ cells per HPF. The distribution in B cell was not significantly associated with graft survival (*p* = 0.13). However, patients with increased numbers of B cells in the TI compartment had the tendency to have DSA present in the serum at time of biopsy. However, this was not statistically significant (*p* = 0.078).

## Discussion

No detailed description on inflammatory cells in renal allograft biopsies showing c-aABMR is currently available. Through multiplex IF staining, we evaluated inflammatory cell presence in glomeruli and TI compartment and related their presence to renal allograft survival. Our study is the first detailed report on the localization and immunophenotypic composition of inflammatory cells in c-aABMR of the renal allograft. Most notably T cells and macrophages were observed within the glomeruli and/or in the TI compartment. Of interest is the increased presence of FoxP3^+^ T cells in the TI compartment, which is significantly associated with inferior allograft survival.

Renal biopsies showing c-aABMR demonstrated vast areas of inflammatory cells denoting the presence of an ongoing immune-mediated process. Substantial numbers of T cells and macrophages were present in the glomeruli and TI compartment.

The glomerular compartment predominantly comprised of CD8^+^ cytotoxic T cells (granzyme B^+^ and CD57^+^) and M2 macrophages (CD68^+^ and CD163^+^), but there was a relatively low amount of single positive CD57^+^ NK cells in the glomeruli. Previous reports have provided evidence for the presence of NK cell transcriptomes marking NK cells as an important player in the pathogenesis of c-aABMR (3, 18, 19). Our study using multiplex IF staining could not confirm the presence of these cells as only a small number of NK cells were present in allografts within the setting of c-aABMR. However, Parkes et al. previously demonstrated that over 50% of increased transcripts in CD16a-activated NK cells are also increased in activated CD8^+^ T cells. The NK and T cells shared transcripts for effector cytokines, chemokines, and other molecules related to effector cell function (18). Of interest was the presence of CD3^+^CD57^+^ T cells in the glomeruli. Several biopsies showed infiltrates of CD3^+^CD57^+^ T cells, and their presence accounted for 6.7% of CD3^+^ cells in the glomerulus. Although these cells have not been further phenotyped, they might indicate the presence of terminally differentiated effector CD8^+^CD57^+^ cytotoxic T cell, possessing NK cell-like properties. These cells are known to have strong cytotoxic potential with high expression of both granzyme B and perforin. The expression of these cytolytic molecules is considerably higher in CD8^+^CD57^+^ T cells than in their CD57^−^ counterpart (20). This type of T cell is presumed to increase in frequency with chronic immune activation as well as during normal aging (21–23). In addition, Björkström et al. proposed that in the setting of chronic immune activation terminal, CD8^+^ T cells can acquire the ability to express FcγRIIIA (CD16) (24). CD16 expressed on CD8^+^ T cells functioned independently from the T-cell receptor and was able to generate effector cytokine production as well as degranulation. CD16 function has been well-studied on NK cells and is best characterized for its role in antibody-dependent cellular cytotoxicity (25). The presence of these cells in combination with the high percentage of granzyme B^+^ T cells found in the glomeruli suggest possible severe cytotoxic damaging effects in the glomerular compartment (26, 27).

The glomerular compartment also contained high numbers of macrophages with widespread distribution in number of CD68^+^ cells per glomerulus. This finding is in accordance with previous literature in which macrophages were significantly associated with clinical rejection and severity, regardless of type of rejection (5–7). Although not statistically significant, the increased presence of macrophages in the TI compartment showed a possible association with graft survival. In addition, it was van den Bosch et al. who provided evidence for increased glomerular infiltration with CD68^+^CD163^+^ macrophages in c-aABMR compared to ABMR and TCMR (7). In accordance with this finding, CD68^+^CD163^+^ macrophages were the predominant subset in the glomeruli (68%), contrary to the TI compartment (39%).

T cells and macrophages were also the main inflammatory cells in the TI compartment but with a different immunophenotypic distribution. The predominant T cells were either CD4^+^ or CD8^+^ T cells and of interest was the observation that, in contrast to the glomeruli, relatively few CD8^+^ T cells expressed granzyme and/or CD57. The areas of the TI compartment with diffuse cellular infiltrates could suggest a possible contributing role for T cells in c-aABMR. Previously, Mengel et al. identified the importance of inflammatory cell infiltrates in protocol biopsies, specifically in areas of IFTA (8). These areas are now classified as i-IFTA/t-IFTA and have also been incorporated into the latest Banff meeting report (2). Although we did not find an association for T-cell infiltrates with (inferior) graft survival, further study is needed to assess if i-IFTA and t-IFTA play an important role in the outcome of graft with c-aABMR.

Of interest in the TI compartment are the CD3^+^FoxP3^+^ cells. We demonstrated that patients with increased FoxP3^+^ Tregs in the TI compartment had inferior renal allograft survival compared to those with few Tregs in the TI compartment. Initially, FoxP3 was identified as the transcription molecule required for Treg development (28). Its presence in certain autoimmune diseases suggests an important role in immune tolerance (29, 30). Similarly, several studies regarding transplantation have shown upregulation of FoxP3 during episodes of acute rejection (15, 31, 32). However, the significance of FoxP3^+^ T cell presence remains uncertain as these studies have shown conflicting data on the relation with graft survival (15, 33–35). For instance, Yapici et al. and Veronese et al. found a correlation between decreased graft survival and an increased density of Foxp3-expressing cells in acute rejection (35, 36).

Our data showing increased presence of FoxP3^+^ Tregs in c-aABMR might be interpreted as an end result of widespread T-cell activation, rather than the cause of poor allograft survival (34). This is in accordance with Bunnag et al., who presented FoxP3 presence as a time-dependent feature reflecting chronic inflammation and showed association with IFTA, rather than FoxP3 as an independent predictor of outcome (34).

In addition, the association with inferior graft survival might be related to the potential of these FoxP3^+^ T cell to easily differentiate into interleukin (IL)-17 producing T helper 17 (Th17) cells, as previously demonstrated in other autoimmune-mediated inflammatory diseases (37). Under normal circumstances, the transcription of FoxP3 in T cells is mainly induced by transformation growth factor-β. However, under proinflammatory conditions involving IL-6, believed to be present in renal allografts during rejection, T cells from a regulatory pathway can be adapted to an inflammatory pathway resulting in Th17 (38, 39). Th17 possess highly proinflammatory capacities rather than immune-regulatory functions.

This is the first study of this nature, describing inflammatory cell presence in glomeruli and the TI compartment of patients with c-aABMR. However, it does have a small number of samples. The presented data are of great interest and provide further insight into the possible pathogenesis of c-aABMR.

In conclusion, the predominant renal-infiltrating immune cells in renal biopsies of c-aABMR are CD8^+^ T cells and M2 type macrophages in both the glomeruli and the TI compartment. Interestingly, in c-aABMR, increased numbers of FoxP3-expressing T cells are significantly associated with poor renal allograft survival.

## Data Availability Statement

The raw data supporting the conclusion of this article will be made available upon request. Requests to access the datasets should be directed to Kasia A. Sablik, k.sablik@erasmusmc.nl.

## Ethics Statement

The studies involving human participants were reviewed and approved by MEC-2019-0308. Written informed consent for participation was not required for this study in accordance with the national legislation and the institutional requirements.

## Author Contributions

KS: conception and design of the work, acquisition, analysis, and interpretation of data, and writing of the manuscript. EJ: conception of the work, acquisition and analysis of data, and critical revising of the manuscript. NP: acquisition and analysis of data and critical revising of the manuscript. MC: conception and design of the work, acquisition of data, and writing, and critical revising of the manuscript. MB: conception and design of the work, analysis and interpretation of data, and writing and critical revising of the manuscript.

### Conflict of Interest

The authors declare that the research was conducted in the absence of any commercial or financial relationships that could be construed as a potential conflict of interest.
